# How and Why Patient Concerns Influence Pain Reporting: A Qualitative Analysis of Personal Accounts and Perceptions of Others’ Use of Numerical Pain Scales

**DOI:** 10.3389/fpsyg.2021.663890

**Published:** 2021-07-02

**Authors:** Brandon L. Boring, Kaitlyn T. Walsh, Namrata Nanavaty, Brandon W. Ng, Vani A. Mathur

**Affiliations:** ^1^Department of Psychological and Brain Sciences, Texas A&M University, College Station, TX, United States; ^2^Department of Psychology, University of Richmond, Richmond, VA, United States; ^3^Texas A&M Institute for Neuroscience, College Station, TX, United States

**Keywords:** pain rating, interpretation of pain, clinical reporting, self-report, attribution bias

## Abstract

Complex factors influence how people report and interpret numerical pain ratings. Such variability can introduce noise and systematic bias into clinical pain assessment. Identification of factors that influence self-rated pain and its interpretation by others may bolster utility of these scales. In this qualitative study, 338 participants described motivations for modulating their own pain reports relative to a numerical pain scale (0–10), as well as perceptions of others’ pain reporting modulation. Responses indicated that people over-report pain to enhance provider belief/responsiveness or the likelihood of pain relief, and out of fear of future pain or potential illness. Concerns of how one’s pain affects and is perceived by others, and financial concerns motivated pain under-reporting. Unprompted, many participants reported never modulating their pain ratings, citing trust in providers and personal ethics. Similar reasons were assumed to motivate others’ pain ratings. However, participants often attributed others’ over-reporting to internal causes, and their own to external. This bias may underlie common assumptions that patients over-report pain for nefarious reasons, distort interpretation of pain reports, and contribute to pain invalidation. Recognition of patient concerns and one’s own personal biases toward others’ pain reporting may improve patient-provider trust and support precision of numerical pain ratings.

## Introduction

Clinical pain assessment relies upon self-report measures – often using numerical pain ratings. These measures are valid, reliable, and easy to use (Hjermstad et al., 2011); however, numerical pain ratings fail to convey the complex nature of the pain experience (Wideman et al., 2019), and some researchers have questioned the unidimensional nature and utility of pain ratings used in isolation (Schiavenato and Craig, 2010; Gordon, 2015; Twycross et al., 2015). Despite established validity, patient numerical pain ratings are often not believed. Clinicians and laypeople alike tend to think that people are in less pain than they report (Guru and Dubinsky, 2000; Marquié et al., 2003; Panda et al., 2006) though this varies across contexts (e.g., based on the demographics of the observer and/or person in pain) (Robinson and Wise, 2003; Garcia-Munitis et al., 2006; Mathur et al., 2014; Ng et al., 2019). This discordance may be related to clinician concerns about how patients differentially use these scales, and patient concerns about how pain reporting influences clinician perception and treatment (e.g., that one will be labeled a drug seeker; Buchman et al., 2016). Therefore, there is a need for greater understanding of how interpersonal and contextual factors potentially influence both the accuracy of numerical pain ratings, and use of pain ratings in clinical decision-making.

Due to the subjective and holistic nature of pain, how and why people report pain is contingent upon their current motivations, contexts, and experience of pain. The construct of pain integrates sensory and emotional experiences (Merskey and Bogduk, 1994), and numerical scales themselves are anchored upon personal experiences and conceptions of pain (i.e., “worst pain imaginable”) (Bergh et al., 2008). As such, it is imperative to explore the potential range of personal factors that guide the use of these scales. Pain reporting in general may also be influenced by internal factors such as negative affect, and contextual factors such as interpersonal trust, expectations of biased physician perceptions and treatment, or an aversion to certain stigmas associated with pain (Koller et al., 1996; Slade et al., 2009; Buchman et al., 2016). Prior qualitative research suggests that pain triggers fears of future pain as well as of one’s pain not being taken seriously (Osborn and Rodham, 2010). Thus, it is possible that individuals may situationally over-rate (e.g., to ensure treatment) or under-rate (e.g., to avoid social judgment) their pain leading to over- or under-treatment of pain, both of which can contribute to unfavorable outcomes (Sherwood et al., 2000; Deyo et al., 2009; Berglund et al., 2012; Kaye et al., 2014). However, with the exception of one study that found that older adults may under-report pain because they believe it is a natural part of aging, little is known about the scope of individual perspectives and concerns that impact pain reporting via numerical pain scales (Kaye et al., 2014).

Beliefs about how others report pain – including suspicion of the person reporting pain, cultural/racial stereotypes, and implicit biases – affect perceptions of others’ pain, and contribute to unfavorable pain treatment decisions and patient outcomes (van Ryn et al., 2011; Sabin and Greenwald, 2012; Wandner et al., 2012). Previous studies indicate that health care providers tend to think patients are in less pain than they report (Guru and Dubinsky, 2000; Marquié et al., 2003). Implicit biases (Staton et al., 2007; Anastas et al., 2020) and attribution of higher pain reports to drug-seeking motivations (Buchman et al., 2016) may compound these effects and subsequently contribute to pain treatment disparities. Together, these perceptions influence treatment decisions that can negatively impact patients’ health and well-being (Bartfield et al., 1997; Woo et al., 2017) and likely reinforce pain stigma more generally (Goldberg, 2017). However, open-ended assessment of more general lay-beliefs about others’ pain reporting motivations is currently missing from the literature.

Identification of the motivations involved in pain reporting behavior through the use of numerical pain ratings and assumptions of the pain reporting motives of others is expected to support increased precision and utility of these scales. However, the varied nature of these motivations has not been firmly established in prior literature – most likely due to the inherent heterogeneity that is difficult to capture with quantitative measures. Further, prior qualitative studies have been constrained to samples of people with a shared clinical diagnosis, and have not assessed common cultural norms and behaviors within the general population. Therefore, in this exploratory study, we examined self-reported motivations for why people over- and/or under-report their own pain, as well as their perceptions of others’ pain reporting motivations.

## Materials and Methods

### Study Design

Data presented here were part of a larger study exploring pain reporting tendencies. This study was conducted online and all participant responses were anonymous and submitted electronically. Self-report methods were used, and no direct interaction between the researchers and the participants occurred. Participants were shown and told to use a numerical pain scale for reference, with 0 representing no pain and 10 representing the worst pain imaginable, and asked whether they typically report a number higher, lower, or equal to what they are actually experiencing. Participants were then asked two open-ended questions, one regarding their own pain reporting motivations (Self: “If you have ever over- or under-reported your pain in real life, please explain why. There are no right or wrong answers, and all responses will remain anonymous and confidential.”), as well as their beliefs about others’ reporting tendencies (Other: “In general, why do you think other people may over- or under-report their pain?”). Participants then provided demographics and were debriefed.

### Participants

We recruited two diverse samples of participants outside of a clinical context, in order to obtain a representative sample of nuanced and perhaps previously unidentified viewpoints related to pain reporting. The Texas A&M University Institutional Review Board reviewed and approved this study and classified it as exempt. Before beginning the survey, all participants were presented with an information sheet outlining the general purpose of the study and electronically confirmed consent to participate. After completion of the survey, participants were electronically debriefed. As participation was voluntary, there was no participant dropout. No follow-up or further interaction occurred with the participants.

#### Sample 1

Sample 1 (S1) included 90 (48 female, 40 male, 1 self-identified as “other”, and 1 declined to answer; 54 White/European American, 17 Latinx/Hispanic American, 1 Black/African American, 8 Asian/Asian American, 3 American Indian/Alaskan Native, 3 multiracial, 1 self-identified as “other”, 2 prefer not to say, and 1 declined answer; ranged between 18 and 22 years old, *M* = 19.40, *SD* = 0.95) student volunteers who received course credit for their participation. All interested volunteers were eligible to participate.

#### Sample 2

Sample 2 (S2) included 248 participants (133 female, 115 male; 129 White/European American, 103 Latinx/Hispanic American, 4 Black/African American, 2 Asian/Asian American, 4 American Indian/Alaskan Native, 5 multiracial, 1 prefer not to say; ranged between 18 and 83 years old, *M* = 46.77, *SD* = 17.61). Participants were recruited and compensated using TurkPrime’s (now CloudResearch) Panels^1^ (Litman et al., 2016; we recently described the advantages of this recruitment method in Mathur et al., 2020). TurkPrime enhances sample representativeness of online samples, has been shown to closely represent the demographic and geographic diversity of the US, and was selected to provide a diverse sample of Americans (Heen et al., 2014; Litman et al., 2016). Participants were paid between $1.75 and $3.11 (depending on the panel and in accordance with TurkPrime standard costs) for completing the brief survey. The survey was open to adult residents of the United States with a minimum quota (*n* = 100) specified to ensure a representative sample of Latinx American participants based on goals of the parent study. Participants were permitted to take as much time as desired, but the majority of participants completed the survey within 20 min.

### Missing Data

All usable qualitative reports are included. In S1, five participants, and in S2, two participants left responses blank to the “self” question. Only one participant from each sample chose not to respond to the question about others’ motivations. In line with current recommendations (Chmielewski and Kucker, 2020), we also screened our data for unrelated or unusual comments. Only three responses to the “self” question, and three to the “other” question were identified as unrelated or uninterpretable (e.g., posting a web address or entering a string of letters) – all from Sample 2, and from the same three individuals – and were removed before analysis.

### Research Team

The research team included three researchers who identify as female (one Multiracial, one South Asian American, and one White American) and two as male (one Asian American, one White American). The authors are all pain researchers (two Ph.D’s, one M.A., one M.S., and one B.S.) within the university system, spanning clinical psychology, social psychology, and neuroscience. The authors are interested in understanding individual factors that contribute to pain experiences, and recognize that they may be biased toward supporting the concerns of the person as a patient. However, the analysis as described below was enacted to reduce the influence of individual researcher bias.

### Analysis

An iterative process of thematic analysis was used to identify qualitative themes in the data following the guidelines for conducting trustworthy thematic analysis outlined by Nowell et al. (2017). In Phase 1 of the thematic analysis, the first and last authors read through the responses multiple times to establish a comprehensive familiarity with the data. These same authors then generated initial categories of pain reporting motivations (i.e., over-reporting, under-reporting, and reporting as is) as part of Phase 2. In Phase 3, these categories were then probed for overarching themes, with some themes being further refined and divided into subthemes following thorough review and reassessment during Phase 4. To ensure equal representation of participant experiences, maximize the generalizability of our findings, and to capture the full scope of reasons behind pain rating modulation, themes were established regardless of the extent of the endorsement of that theme (rather than ignore them or relegate them to an amorphous “other” category). The first and last author then crafted succinct and appropriate names for all themes/subthemes in Phase 5. Finally, in Phase 6, two independent coders (second and third authors) previously blind to the data and theme development process then assigned these themes/subthemes to each participant response; these were subsequently compared for consistency. Any remaining discrepancies were discussed and resolved by the authors until 100% agreement was achieved. All authors then worked together to write the descriptions of the themes within the report, making sure to include quotes that accurately supported the themes being described. Table 1 was also created to provide an overview of the themes/subthemes, as well as to present the frequency of each endorsement.

**TABLE 1:** Qualitative themes.

**Theme/*Subtheme***		**Sample 1 (S1, *n* = 90)**	**Sample 2 (S2, *n* = 248)**	**Combined sample (*n* = 338)**
***Self-perspective: reasons for under-reporting pain***				
Impression management		9	13	22
	*Appear tough*	7	5	12
	*Self-conscious/embarrassed*	4	9	13
	*Adjustment to expectations*	0	1	1
Downplaying pain		8	4	12
Fear and avoidance		10	20	30
	*Fear of bad news/denial*	1	1	2
	*Avoidance of medical settings*	1	5	6
	*Avoid medication/treatment*	5	13	18
	*Reduce time in care*	3	4	7
Cost		7	2	9
	*Cost/access to care*	1	1	2
	*Reduce repercussions*	5	1	6
	*Price of desired outcome*	1	0	1
Other-oriented		4	8	12
	*Concern for social networks*	2	5	7
	*Practitioner inconvenience*	2	1	3
	*General concern*	0	2	2
Cognitive modulation		3	1	4
	*Counteract over-reporting*	1	1	2
	*Subjective misunderstanding*	2	1	3
Deference/doctor as expert		1	1	2
Simply restating		1	2	3
	*Total endorsements*	78	101	179
	*Total participants that endorsed*	33	46	79
***Self-perspective: reasons for over-reporting pain***				
Ensure treatment		19	28	47
	*Taken seriously/secure assistance*	12	14	26
	*Receive medication/treatment*	10	9	19
	*Accelerate care*	1	9	10
	*Discrimination*	1	0	1
Solicit social support		1	1	2
Alternative motives		6	9	15
	*Influence of addiction*	0	1	1
	*Extra/stronger medication or treatment*	5	6	11
	*Avoid work/social interaction*	1	2	3
Fear and avoidance		4	8	12
	*General fear/anxiety*	1	3	4
	*Proactive reporting*	3	5	8
Cognitive modulation		1	5	6
	*Prior/lack of experience*	1	3	4
	*Dynamic appraisal*	0	2	2
Justification		2	0	2
Simply restating		2	2	4
	*Total endorsements*	70	107	177
	*Total participants that endorsed*	30	47	77
***Self-perspective: reasons for neither under- nor over-reporting pain***				
Trust in doctor		1	2	3
Ensure proper treatment		2	10	12
Morals		2	15	17
Simply restating		16	78	94
Individual differences		0	3	3
	*Total endorsements*	21	108	129
	*Total participants that endorsed*	21	101	122
***Other perspective: reasons for under-reporting pain***				
Impression management		22	34	56
	*Appear tough*	16	21	37
	*Self-conscious/embarrassed*	8	16	24
	*Deflect drug-seeking stereotypes*	0	1	1
	*Conveying health/resilience*	0	1	1
Downplaying pain		8	10	18
Fear and avoidance		21	28	49
	*Fear of bad news/denial*	10	7	17
	*Avoidance of doctors/medical settings*	2	7	9
	*Avoid medication/treatment*	7	16	23
	*Reduce time in care*	1	3	4
Cost		4	10	14
	*Cost/access to care*	2	7	9
	*Reduce repercussions*	2	2	4
	*Time*	1	1	2
Other-oriented		6	6	12
	*Concern for social networks*	3	1	4
	*Practitioner inconvenience*	2	0	2
	*Concern for un-named other*	2	5	7
Cognitive modulation		2	3	5
	*Pain expectations/misconceptions*	0	1	1
	*Prior/lack of experience*	0	1	1
	*Subjective misunderstanding*	2	1	3
Deference/doctor as expert		1	0	1
Enjoy pain		0	1	1
Simply restating		1	3	4
	*Total endorsements*	123	186	309
	*Total participants that endorsed*	50	81	131
***Other perspective: reasons for over-reporting pain***				
Ensure treatment		32	71	103
	*Taken seriously/secure assistance*	15	24	39
	*Receive medication/treatment*	16	47	63
	*Accelerate care*	6	15	21
Solicit social support		1	7	8
Derogatory assessment		7	12	19
Alternative motives		17	66	83
	*Influence of addiction*	2	9	11
	*Extra/Stronger medication or treatment*	8	46	54
	*Receive compensation*	0	1	1
	*Psychosocial*	7	15	22
Fear and avoidance		1	3	4
	*General fear/anxiety*	0	1	1
	*Proactive reporting*	1	1	2
Cognitive modulation		2	7	9
	*Prior/lack of experience*	0	2	2
	*Adjustment to expectations*	1	2	3
	*Subjective misunderstanding*	0	4	4
Justification		2	0	2
Simply restating		3	5	8
	*Total endorsements*	121	338	459
	*Total participants that endorsed*	58	138	196
***Other perspective: reasons for neither under- nor over-reporting pain***				
Ensure proper treatment		0	1	1
	*Total endorsements*	0	1	1
	*Total participants that endorsed*	0	1	1
***Other perspective: recognition of pain subjectivity***				
Individual differences		1	4	5
	*Total endorsements*	1	4	5
	*Total participants that endorsed*	1	4	5

## Results

For readability, and due to similarities across samples, results are presented together in the narrative. The sample from which the quote was taken is indicated by S1 (undergraduate student sample) or S2 (TurkPrime sample). Prevalence of themes and sample comparison are presented in Table 1.

### Self

Multiple common themes (many broken down into further subthemes) arose for why participants had previously under- or over-reported their pain in clinical settings. Unexpectedly, participants also frequently reported a third option to the question about one’s own pain reporting – noting that they have never over- or under-reported their pain.

#### Self-Under

##### 1. Impression management

A common theme that participants endorsed was concern for how they or their pain would be perceived by others. Participants often cited that this concern led to under-reporting their pain in efforts to manage others’ perceptions or judgments.

###### 1.i. Appear tough

Belief that showing pain would be perceived as weakness was frequently reported as to why participants under-reported pain. Participants often felt that if they reported the amount of pain they were actually experiencing, they might be considered weak and thus under-reported to appear strong or tough [*“I have under-reported my pain before in order to seem tougher.” (S1); “*… *because I thought if I said how much pain I was in I would be considered weak” (S1)*].

###### 1.ii. Self-conscious/embarrassed

Others expressed worry about appearing dramatic as well as experiencing embarrassment about reporting their actual amount of pain. Thus, the participants under-reported to prevent or reduce either feelings of embarrassment and/or perceptions that they were unnecessarily exaggerating their level of pain [*“*… *I don’t want to sound too dramatic*…*” (S2); “Embarrassed to admit the level of pain” (S2)*].

###### 1.iii. Adjustment to others’ expectations

One individual stated that their under-reporting was influenced by what they perceived others’ expectations or beliefs about what their pain should be [*“*… *because I thought that saying anything else would seem out of the normal, as I was told that I should not still be feeling pain when I was” (S2)*].

##### 2. Downplaying pain

Some participants mentioned that they tended to under-report their pain in order to minimize urgency when they thought their pain was not personally concerning [*“I will under report something if the situation is not that serious.” (S1); “i do when i*… *just don’t think there is a real problem but my wife forces me to the hospital” (S2)*]. Others believed that they did not need additional help and could manage the pain themselves [*“I have under-reported the level of pain I was experiencing because I thought I could handle it.” (S1)*].

##### 3. Fear/avoidance

Participants also disclosed having under-reported their pain due to fears or avoidance of some facet of their pain experience. Whether specific to the pain itself or more generalized to the health care system, participants adjusted their pain ratings to put off undesired outcomes.

###### 3.i. Fear of bad news/denial

Participants expressed a fear and avoidance of being given a diagnosis with implications greater than the subjective pain itself, and therefore were motivated to downplay their situation by under-reporting their pain [“*Yes, I tend to underreport my pain, I never want to hear the doctor say I have something terribly wrong with me*” *(S1)*].

###### 3.ii. Avoidance of medical settings

Some participants admitted to under-reporting their pain to avoid entering health care settings [“*sometimes I low ball it, so I don’t have to go to the doctor*” *(S2)*], or once there, prevent further clinical intervention [“*sometimes I do in fear I will be admitted to the hospital*” *(S2)*].

###### 3.iii. Avoid medication/treatment

Other participants, having actively sought medical care, admitted to under-reporting their pain to avoid medications, particularly pain killers [“*I tend to report pain to the same scale or less than my actual pain because I tend to avoid high doses of pain killers*” *(S1)*] and/or treatments and procedures [“*Underreported severity of pain in order to avoid any additional treatment*” *(S2)*].

###### 3.iv. Reduce time in care

Under-reporting was also endorsed as a means to hasten participants’ exit from the medical setting [“*I feel like I have underreported pain or other symptoms because I just want to leave the appointment*” *(S1)*]. For some, this was predicated on instances where others had convinced or coerced them to seek care in the first place [“*Sometimes if I am not in that much pain but somebody makes me go to the doctor I will say my pain is less because I want to leave*” *(S1)*].

##### 4. Cost

Another motivator behind why participants under-reported their pain was the costs associated with divulging that pain. This included both monetary concerns as well as the desire to limit repercussions affecting other responsibilities that might result from reporting one’s actual pain level.

###### 4.i. Cost/access to care

The price of seeking and receiving treatment was cited as one reason for under-reporting pain [“*I don’t want expensive medical procedures done that’s [sic] are unnecessary*” *(S1)*].

###### 4.ii. Reduce repercussions

Other costs were more abstract, such as not wanting to miss out on limited experiences or shirking obligations such as sports, school, or work [“*I have under-reported pain in my life during volleyball games to continue playing even though I am injured*” *(S1)*].

###### 4.iii. Price of desired outcome

One participant stated that they under-report their pain in certain situations because of a perceived association between pain and a desired outcome [“*I under report pain in the dentist office because I know the procedures are to make my teeth look pretty and I generally correspond beauty with pain*” *(S1)*].

##### 5. Other-oriented

Individuals often reported under-reporting pain due to consideration of others, both within their personal lives and professional contexts.

###### 5.i. Concern for social networks

Specifically, participants identified concern for those within their social networks (i.e., family/friends) as a reason to under-report pain. Individuals reported not wanting their loved ones to stress, worry, or be too concerned about their pain [*“*… *I was with my mom and did not want to worry her” (S1)*]. Others cited broader social networks that may be impacted by practical consequences of reporting pain rather than the emotional response to pain reporting itself. Rather than concern for the worry or stress pain-reporting may provoke, these participants cited the consequent ‘suffering’ or loss others may feel due to the implications of reporting pain [*“*… *when I know that someone else will suffer if I don’t perform*…. *Even if I had pain, I kept performing or playing for the benefit of the team*…*” (S1)*].

###### 5.ii. Practitioner inconvenience

Furthermore, participants also considered the inconvenience their pain may cause healthcare providers. Though healthcare providers are likely to be outside of the individual’s social network, similarly inhibiting pain-reporting behavior was reported. Rather than concern for the emotional and perhaps affectionate response of family and friends, some participants stated they have under-reported pain to avoid frustration or inconvenience on the physician’s part [*“Sometimes you feel like it may be too much trouble for the doctor” (S1); “Under reported cause depending on doctor seeing me it seems like they have better things to do than to tend to someone in pain” (S2)*].

###### 5.iii. General concern

Two participants endorsed that they would under-report pain out of concern for the way others would respond – either sympathetically or as if the respondent is a bother/burden. These participants did not provide further information regarding who specifically they are considering [*“*…*I don’t want sympathy” (S2); “I have under report pain because I felt like I was being a bother” (S2)*].

##### 6. Cognitive modulation

Some individuals reported altering their pain ratings following a cognitive reflection of their pain, how their pain might be perceived, or how they think they *should* be reporting their pain.

###### 6.i. Counteract over-reporting

The majority of responses within this theme included reports of lowering pain ratings due to awareness of over-reporting tendencies that they are attempting to counteract [*“I usually think I’m [sic] too sensitive to the pain or overreacting, so I would report a lower number” (S2); “Often times, I feel that if I am given attention for my complaints, I am subject to subconsciously rate my pain higher than it actually is. Therefore, I give a “low-ball” rate of my pain to counter this bias” (S1)*].

###### 6.ii. Subjective misunderstanding

Other participants misunder- stood the subjective nature of pain and thought of their pain in relation to some objective standard. Some participants believed that their pain tolerance was higher relative to others, and therefore the rating that they provided was lower than what others would provide [“*I usually under report as I have an overall higher pain tolerance” (S1)*], or felt as though they need to adjust their rating due to the fluctuation of the pain they experienced over time [*“I usually under-report my pain. because by the time I go to the doctor the pain has already lessened in comparison to the previous day(s)” (S1)*].

##### 7. Deference/doctor as expert

Two participant responses also revealed a tendency to view the physician’s expectations or evaluations as more credible than their own pain perceptions [*“I have under reported my pain in real life because I feel like the doctor knows more than I do about what is wrong with me and knows that it does’nt [sic] actually hurt as much as I am saying or they won’t believe me” (S1)*].

##### 8. Simply restating

A few individuals restated that they have under-reported pain before but gave no further reasoning as to why they under-reported their pain [*“If anything I’ve under reported” (S2); “Yes I have said I’m ok but was in pain more than I showed” (S2)*].

#### Self-Over

##### 1. Ensure treatment

Many participants mentioned that ensuring proper care or treatment from their provider was an important motivator for over-reporting their pain. Participants expressed concern for being under-treated for their pain and as a result, over-reported their pain to receive thorough and proper care from their provider.

###### 1.i. Taken seriously/secure assistance

Some participants expressed worry for not being taken seriously and or distrust in their doctor to provide thorough care. Thus, participants over-reported their pain in order to be believed and to secure thorough treatment and care [*“I often report a higher level of pain due to a fear of not being taken seriously enough” (S1); “so he would take me serious” (S2)*].

###### 1.ii. Receive medication/treatment

Others indicated over-reporting their pain to help their odds of receiving medication or treatment that would relieve their pain [*“I have four herniated disks and get frustrated with continual pain and want to get relief.” (S2); “when i had a tooth ache and i wanted the tooth pulled because it has given trouble [before]” (S2)*].

###### 1.iii. Accelerate care

A few participants over-reported their pain to accelerate the care they received such as relieving their pain quicker, reducing time spent at visits, or limiting the number of visits in the future [*“I’ve over reported pain in the ER so I would be seen faster” (S2); “Over in order to be treated faster” (S2)*].

###### 1.iv. Discrimination

One participant highlighted concern about being taken seriously in the context of discrimination [*“Over pain because being a black women I already feel intimidate and i feel I have to over react to my symptoms so they’ll take me serious” (S1)*].

##### 2. Solicit social support

Participants endorsed that they may over-report their own pain when seeking supportive behavior from those around them. Responses included solicitations of support from friends as well as family members [*“[sometimes] I need more help and support” (S2); “…if I am around my parents and I know they will help me or take care of me, I might over-report a little bit.” (S1)*].

##### 3. Alternative motives

Participants also noted a number of alternative motives that may explain their own behavior of over-reporting their pain. This theme included reasons that they may over-report pain with the intention of gaining or avoiding particular outcomes.

###### 3.i. Influence of addiction

Some participants attributed over-reporting of pain due to addiction or dependence on medications [*“Because I was addicted” (S2)*].

###### 3.ii. Extra/stronger medication/treatment

Aside from the influence of addiction, participants also endorsed seeking stronger or what they believed to be more effective medication for their pain [*“In the hope of getting stronger medicine” (S2); “I might over report pain if it means getting better pain killers.” (S1)*].

###### 3.iii. Avoid work/social interaction

Another sub-theme of motivated over-reporting included a desire to avoid professional or social interpersonal interactions [*“I sometimes over reported a pain so I wouldn’t have to go out” (S2); “In order to get a doctors note*… *I will exaggerate pain” (S1)*].

##### 4. Fear and avoidance

Fear and anxiety about their pain and the desire to avoid future pain were other reasons participants disclosed over-reporting their pain.

###### 4.i. General fear/anxiety

Some of these responses described general fear or worry about the pain they were experiencing [*“If I’m having a procedure done I would exaggerate a little due to fear” (S2); “depending on how scary or how worried you are about a disease, i may…over report my pain. If I’m really worried, i might over report it.” (S2)*].

###### 4.ii. Proactive reporting

Other participants related having over-reported their pain to proactively address any future pain that they expected could occur, whether it be during a medical procedure [*“I’ve over-reported pain during procedures during which the anesthetics have begun to wear off. I did this so that the doctors would see my pain as a priority and immediately try to rectify the mistake, as opposed to ignoring it and continuing with the procedure” (S1)*], or in the time following treatment [*“I slightly over-reported my jaw pain. I did this with the intentions of knowing that the pain was going to get worse as time progressed so my thought was that I was being proactive” (S1)*].

##### 5. Cognitive modulation

Participants at times reported cognitively analyzing interference with their pain experience in relation to their own as well as others’ expectations of pain and changing their pain rating accordingly.

###### 5.i. Prior (or lack of) experience

Some participants retrospec- tively took into account their lack of experience in pain reporting as a potential reason to over-report their pain [*“perhaps as a child i over rated pain not knowing how to tryly [sic] evaluate” (S2); “Looking back, there was an instance where I over-reported the pain I was feeling. However, it was when I was young and didn’t have a solid grasp on what the ends of the scale might represent” (S1)*].

###### 5.ii. Dynamic appraisal

Other participants adjusted their ratings due to the dynamic nature of their pain experience [“*I have reported (over) as the pain would come and go.”; “I sometimes will over rate my pain because I feel uncomfortable” (S2)*].

##### 6. Justification

Participants expressed over-reporting their pain to justify their treatment seeking, whether this be to allay their own doubts [*“The only times I have over reported pain is when I felt like I was being dramatic for coming to the doctor so would want to justify being there. Nothing crazy, but maybe like one number higher on a scale of 1–10.” (S1)*], or to make sure they received adequate compensation for their costs of receiving treatment [*“Health care in the United States is always paid in one way or another and usually I personally like getting my ‘money’s worth,’ so I would over report my pain if I feel like it is not actually high enough to be treated just because I would want to be prescribed some sort of medication so that that same pain can go away or in other words, be treated” (S1)*].

##### 7. Simply restating

Several participants restated or gave instances when they over-reported their pain but did not give an explanation or go into further detail about why they over-reported [*“*… *I have also overrated pain such as getting a filling at the dentist.” (S1); “I probably over-reported pain during my first labor*…*” (S2)*].

#### Self-Neither

A considerable number of participants indicated that they neither under- nor over-report their pain, despite this not being directly asked of them within the question. Whether it was due to trust in the healthcare provider, ensuring proper treatment, their underlying morals, or expressing individual differences in the pain experience, participants disclosed their tendency to accurately report their pain.

##### 1. Trust in doctor

Participants indicated that they had reported their actual pain level due to trust in the doctor [*“If I feel like I’m being given their full attention and they’ve listened to all my concerns, I’m okay to be truthful and accept if they don’t think anything is wrong” (S1)*].

##### 2. Ensure proper treatment

Other participants indicated that they neither over- nor under-report their pain to ensure that they were treated accurately [*“No. I try to be as honest as I can when it comes to dealing with doctors or nurses. I want to be treated for what I actually am experiencing I do not fake my pain or symptoms” (S2)*], particularly due to concerns that by reporting otherwise might impact their health further or lead to unwanted outcomes [*“No. Why would I? Under reporting it would allow you to keep hurting, over reporting might lead to being given too much pain relieving meds” (S2)*].

##### 3. Morals

Many participants endorsed a lack of over- or under-reporting in terms of their own pain due to a moral or virtuous commitment to honesty [*“I have not because I feel the need to be honest.” (S1); “…I take pain very seriously and would not lie” (S2)*].

##### 4. Individual differences

Participants also alluded to individual differences in pain sensitivity as a reason to not under- or over-report pain [*“No, everyone’s pain level is different” (S2); “I [don’t] think so, but the amount of pain someone can endure is different among people. One type of pain might be considered bearable, but a different type of pain might seem either easy or harder to for you to endure” (S2)*].

##### 5. Simply restating

Other participants stated that they have never under- or over-reported their pain but did not give an explanation [*“I have never done either of these” (S1); “I usually do not over or under, I just say what I am feeling” (S1)*].

### Other

Participants’ perception of why others might modulate their pain ratings in clinical settings produced similar responses as to those given for the self for under- and over-reporting, although over-reporting was more often attributed to negative assumptions of the motives of others.

#### Other-Under

##### 1. Impression management

One of the more common themes individuals gave for why other people under-report pain was the concern that they would be viewed negatively or they felt self-conscious about their pain. Individuals believed others would under-report to avoid negative perceptions or to lessen self-conscious emotions they may have about their pain.

###### 1.i. Appear tough

Similar to themselves, participants frequently speculated that others would under-report their pain so that they would appear strong or to not be seen as weak [*“People under-report pain because they do not want other people to view them as weak or not tough.” (S1); “I think many people underrate their pain for the same reason. Many people do not want to appear as if they are weak and have a strong self-image” (S1)*].

###### 1.ii. Self-conscious/embarrassed/“not appear dramatic”

Partici- pants also reasoned that others may feel self-conscious about their pain and would under-report their pain to mitigate feelings of embarrassment or to not be perceived as exaggerating their pain [*“People also may underrate their pain if they don’t think their issue is a big deal, so they don’t want to make a scene about it.” (S1); “Because they are afraid of what the Dr may think of them” (S2)*].

###### 1.iii. Deflect drug-seeking stereotypes

Some participants gave additional reasons for why others might under-report pain that were also associated with concerns for others’ perceptions. One participant stated that people under-reported so that they didn’t arouse suspicion for seeking drugs [*“They want drugs or don’t want to seem like thats all they want” (S2)*].

###### 1.iv. Conveying health/resilience

Another participant thought that in addition to wanting to appear tough, they might also want to appear healthier than they actually are [*“I think sometimes people under-report pain when they want to seem. healthier than they really are.” (S2)*].

##### 2. Downplaying pain

Some participants stated that others may under-report because they feel the pain isn’t that serious and can be managed without additional help from outside sources or because they believe nothing is wrong. In this instance, under-reporting was due to one’s beliefs about their pain and whether it was manageable or to reduce perceptions of severity within the individual or others [*“Most likely to seem as if their condition is minimal in comparison to other conditions” (S1); “If they under report their pain, one possibility is that they don’t really think anything is wrong with themselves” (S1)*].

##### 3. Fear/avoidance

Similar to their own concerns, participants suggested that others might under-report their pain due to fear or avoidance of some aspect of the pain experience, from the general [*“for under people are scared” (S1)*], to more specific subthemes, including fear of bad news/denial, avoidance of doctors/medical settings, avoidance of medicine or treatment, and to avoid further time in care.

###### 3.i. Fear of bad news/denial

Participants provided examples of other’s under-reporting due to fear of receiving outcomes or diagnoses with implications greater than an acute pain episode [*“I assume people under-report their pain because they are afraid of what the outcome could be if the [sic] acknowledge the amount of their pain” (S1)*].

###### 3.ii. Avoidance of doctors/medical settings

Participants also suggested that others might under-report to avoid doctors or medical settings in the first place [*“I think people do this because they are afraid of hospitalization it is an unpleasant experience to them” (S2); “people who underreport I have found out are afraid of doctors” (S2)*].

###### 3.iii Avoidance of medicine or treatment

The participants further posited that others may under-report to avoid either medicine [*“people underreport it because they do not like to take drug” (S2); “I believe people underreport pain to avoid being prescribed pain killers” (S2)*], or other forms of treatment [*“Underreporting, because they don’t want to need treatment or surgery” (S2)*].

###### 3.iv Avoid further time in care

Once having sought treatment, participants thought that others might under-report to avoid staying in a clinical environment [*“Because*… *they are tired of the medical setting” (S2)*].

##### 4. Cost

Participants suggested that others might under-report their pain for monetary reasons to prevent missing out on experiences or obligations, or for the price of one’s time.

###### 4.i. Cost/access to care

Being unable to afford medical care was often described as a reason for why others might under-report their pain [*“It depends, but usually I feel that people under report their pain in order to avoid the cost of medical expenses” (S2); “They might under-estimate if they don’t want to/don’t have the means to get the possible care” (S1)*].

###### 4.ii. Reduce repercussions

Participants also thought that others might under-report to avoid any repercussions that might be associated with divulging pain, whether this be for a limited experience [*“Under report because maybe they were injured from a sport in which they still may want to participate in and they want the doctor’s ok to continue playing even though their pain level my be higher than what they may actually report” (S1)*], or for obligations such as work [*“Under – It could be for work” (S2)*].

###### 4.iii. Time

The time lost by going to seek care was another reason listed for under-reporting pain [*“If you’re annoyed by doctors appointments*… *you might choose to under report your pain.” (S2); “To avoid hassle” (S1)*].

##### 5. Other-oriented

Similar to tendencies reported related to under-reporting of one’s own pain, individuals suggested that other’s may also under-report pain due to a concern for the reactions, worry, or burden of others in their lives.

###### 5.i. Concern for social networks

Some participants referenced a concern for other people as a reason that others may under-report their pain, similar to as they did in the context of the self. Specifically, respondents referenced that others may not want to worry their family members [*“*… *they do not want people to worry about them” (S1); “I think people may underreport their pain because they may not want to worry any family members” (S1)*].

###### 5.ii. Practitioner inconvenience

Aside from family members, individuals also referenced that others may under-report to prevent worrying, inconveniencing, or bothering their practitioners or physicians [*“*… *to not bother the doctors with your actual level of pain” (S1)*].

###### 5.iii. Concern for un-named other

Other respondents mentioned wanting to prevent concern or worry, however, didn’t mention a specific target of concern [*“*… *don’t want to be a pain or complain” (S2); “If they under repertory they [don’t] want to bother or feel like a burden.” (S2)*].

##### 6. Cognitive modulation

###### 6.i. Pain expectations/misconceptions

A few participants reported that others may have cognitive biases or perceptions (that may be rational or irrational) that contribute to under-reporting of pain. These included the idea that under-reporting may be related to ‘wishful thinking’ that the pain will lessen, or conceptions related to what may be expected when reporting pain. Some participants also referenced lack of understanding of how to perceive or report pain as a reason others may under-report pain [“*under report thinking it will make illness less severe” (S2)*].

###### 6.ii. Prior (or lack of) experience

Others suggested that a lack of experience with pain and/or pain reporting may affect pain ratings [*“They may under report their pain in*… *unsureness of how to rate their pain.” (S2)*].

###### 6.iii. Subjective misunderstanding

Some participants thought that the ratings that others provided would be under-reported due to an objective tolerance level, not considering that that rating would be their subjective experience [*“Under-report because they have high pain tolerance” (S1); “people who. have naturally high pain tolerance might under-report pain” (S1)*].

##### 7. Deference/doctor as expert

One individual suggested that others are likely to under-report their pain under circumstances in which their physician has an established perspective on their pain. This explanation touches on an adjustment of individuals’ pain perception, on a conscious or subconscious level, to better align their pain perception with the pain perception of the physician [*“in regards to if the specialist sees that the problem is not as bad as the patient thinks it is, I think other people under-report their pain because since they are seeing someone specialized on the problem they are concerned with, so the opinions of the specialist will influence the patient’s own opinion more*…*” (S1)*].

##### 8. Enjoy pain

One participant suggested that others might under-report their pain due to enjoying the pain in some fashion [“*For those that under-reported they may have some Freudian attraction to masochism*” *(S2)*], suggesting that people might prefer to prolong their pain experience for some derived pleasure.

##### 9. Simply restating

Some restated that others may under-report their pain but gave no further explanation for why this may occur [*“Other people may*… *under report their pain.” (S1); “I think they under report their pain” (S2)*].

#### Other-Over

##### 1. Ensure treatment

Participants reasoned that others over-reported their pain for similar concerns over receiving appropriate care and attention from their healthcare provider.

###### 1.i. Taken seriously/secure assistance

Participants also believed others over-reported their pain because they didn’t trust their doctor to provide satisfactory care or in order for their pain to be believed. Therefore, others over-reported their pain to be taken seriously and to guarantee proper care and treatment from their provider [*“People who over-report want to avoid feeling brushed off or not being taken seriously” (S1)*; *“I think other people could over report because they feel that doctor’s don’t take pain seriously unless it is extreme pain” (S1)*].

###### 1.ii. Receive medication/treatment

Securing medication or treatment to relieve their pain was another reason people believed others would over-report their pain [*“Over to make sure they get medication” (S1)*; *“Over because they want treatment” (S2)*].

###### 1.iii. Accelerate care

Several participants thought others may over-report their pain in order to receive care and be treated more quickly or to prevent future visits to the doctor [*“They overrate it due to a desire to make sure that the problem is handled faster” (S1); “They might over-estimate in order to get as much help as possible or to prevent multiple visits to the doctor” (S1)*].

##### 2. Solicit social support

Similar to reasoning in the ‘self’ perspective, participants thought others may over-report pain to obtain sympathy or care from those around them [*“some people may want more sympathy” (S2); “maybe they*… *want someone to care more about them” (S2)*].

##### 3. Derogatory assessment

Unlike reasons given for the self, some participants attributed others’ over-reporting to character traits deemed to be deficient, both physically [“*They’re not very tough*” *(S1)*], and mentally [“*many people do exaggerate when it comes to pain since many of them can’t handle any hardship and we live in an age where even a little bit of wrong is treated like a national disaster*” *(S2)*].

##### 4. Alternative motives

As seen in the ‘self’ perspective, participants reasoned that others may over-report pain for prevention or achievement of specific consequences.

###### 4.i. Influence of addiction

Participants mentioned that others may be addicted to drugs and may over-report to presumably obtain drugs upon which participants may be dependent [*“Drug addicted” (S2)*].

###### 4.ii. Extra/stronger medication/treatment

As seen in the ‘self’ perspective, participants also mentioned that others may over-report to seek medication that is more powerful or effective, or simply greater quantities of medication [*“They may over report pain because they want more powerful pain medication” (S2); “Some might over report thinking they’ll get something very strong” (S2)*].

###### 4.iii. Psychosocial

Another sub-theme endorsed revolved around participants suggesting others may over-report pain to seek attention from those around them. Unlike the ‘social support’ sub-theme, responses in this sub-theme did not include notions of seeking care or comfort, but rather attention alone [*“Because they seek attention” (S2); “others are prima donnas who just want attention” (S2)*].

##### 5. Fear/avoidance

Participants thought that others might over-report their pain due to fear or anxiety about pain, or to avoid potential future pain.

###### 5.i. General fear/anxiety

Some of this was general fear or anxiety about pain itself or experiences associated with pain [*“Over because they are afraid.” (S2); “Higher…They feel helpless and hopeless.” (S2)*].

###### 5.ii. Proactive

Whereas other participants suggested that others might over-report their pain to proactively address any future pain that may be experienced [“*People might over-report pain if they think the pain might increase at a future time, so they can be medicated for the pain and not have to experience it later*” *(S1)*].

##### 6. Cognitive modulation

A small number of participant responses alluded to a thought process involved in the over-reporting of pain.

###### 6.i. Prior (or lack of) experience

Some of these were attributed to lack of experience in reporting pain [*“I think sometime people may over-report pain because*… *they don’t know what real pain is” (S2); “They may over report their pain*… *[because] they may also be unsure of how to rate their pain” (S2)*].

###### 6.ii. Adjustment to expectations

Participants also mentioned that others may adjust their pain reports based on their pain experience or the perception of others [*“…I think people over-report their pain in agreeing with the specialist” (S1);* “*Some people may over report pain because they assume they’ll be in pain” (S2)*].

###### 6.iii. Subjective misunderstanding

Similar to thoughts about others’ under-reporting, participants used pain tolerance as an objective standard for pain ratings, suggesting that others would over-report due to increased sensitivity to pain [*“I think they might overreport it if they have a low pain tolerance” (S2); “Over reporting, perhaps because*… *they are very sensitive to pain” (S2)*].

##### 7. Justification

Some participants thought that others might over-report their pain to justify seeking treatment, both to confirm their pain to themselves [*“To make themselves feel better, more pain* = *I actually came to the doctor for an actual reason” (S1)*], or to make sure that their money isn’t going to waste [*“To have their money’s worth from that appointment” (S1)*].

##### 8. Simply restating

Similar to reasons given for the self, some participants simply restated that others may over-report their pain but did not indicate why that may be [*“I generally think people over report their pain” (S2); “I think that they generally over report pain” (S2)*].

#### Other-Neither

##### 1. Ensure proper treatment

One participant believed that others would neither over- or under-report their pain due to there being no benefit or utility in doing so [*“they would most likely tell the truth why lie” (S2)*].

#### Other-Recognition of Pain Subjectivity

Finally, some participants – while not providing specific reasons why others might over- or under-report – recognized that there is variability in the ways in which others might present their pain [*“I feel like it varies person to person- there’s no formula” (S2); “I’m not sure each person is different and how they handle their pain would be different” (S2)*].

## Discussion

Pain reporting via numerical scale is often the primary way patients communicate their multifaceted pain experience to their clinical providers. Contextual factors, personal motivations and fears, and interpersonal trust contribute to the use and interpretation of these scales. Present results clarify patient motivations for modulation in clinical contexts and provide insight into cultural conceptions of why others might modulate their pain ratings in similar settings.

Qualitative reports indicate that, while many people endeavor to report their pain as accurately as possible, there are nuanced reasons why people may modulate their pain ratings (i.e., over- or under-report their own pain). The primary reason people over-report their pain is to be taken seriously and ensure adequate attention is given to their pain. Taken together with prior work showing that fear of not having one’s pain believed is a significant concern for many individuals (Upshur et al., 2010) and that the belief one’s pain will not be thoroughly assessed often damages trust of health care providers (Buchman et al., 2017), the current results point to distrust as a potential mediator of elevated pain reports.

Common reasons people gave for under-reporting (and why they think others might under-report) were related to avoiding ‘bad news’ or undesirable medical treatments and impression management – conceptualized as the tendency to be self-conscious regarding the way others may perceive their reports of pain. Worry about potentially distressing information, treatment, or judgment from others appeared to largely drive the tendency to under-report one’s own pain. This suggests that efforts to allay these fears may support more accurate reporting.

Pain stigma emerged as a superordinate motif related to pain modulation across perspectives. Responses revealed cultural conceptions of pain as weakness, engagement in impression management related to pain reports, and stigma consciousness (i.e., self-consciousness about one’s pain, and concern that their pain reports will not be perceived as valid by others). Though the impact of chronic pain stigma is slowly gaining much needed attention (Carr, 2016; De Ruddere and Craig, 2016; Goldberg, 2017) the present results indicate the pervasiveness of the cultural stigma of pain even in its most general conceptualization, and outside of clinical settings or samples.

It is important to note that participants frequently endorsed reporting their pain as is, despite specifically being asked to provide reasons for over- or under-reporting in the research question. Participants cited concerns of detrimental effects of adjusting one’s ratings on the accuracy of treatment, as well as a general desire for honesty. This insight suggests that people may not modulate their pain as frequently as health care providers believe they do; recognizing that many patients report accurately may support future efforts to improve patient-provider trust and decrease negative assumptions about patient motives within the healthcare system which are known to impact assessment and treatment decisions (Tait et al., 2009).

Overall, participants thought that other people were likely motivated by reasons similar to their own to modulate pain ratings. Many responses reflected empathy for the plight of others and provision of the benefit-of-the-doubt (e.g., like me, others might over-report to be believed or receive treatment). However, participants did provide additional reasons why they thought other people might over-report, rather than under-report, pain. These included negatively valenced assumptions about motives that: (1) did not emerge when providing reasons for self-modulation of pain reports; and (2) were not treatment oriented. Furthermore, these reasons were often based on some inherent physical or character deficiencies. That these reasons did not correspond to similar self-related endorsements suggests influence of the fundamental attribution error (i.e., attributing more negative behaviors of others to internal causes, and one’s own behaviors to more external or situational causes) (Jones, 1979). Such beliefs may also contribute to clinical bias and cultural assumptions that patients over-report their pain for nefarious reasons (e.g., to obtain opioids) (Miceli and Katz, 2009), and complement prior studies indicating discordance between patient reports and observer perceptions (Guru and Dubinsky, 2000; Marquié et al., 2003; Panda et al., 2006; Mathur et al., 2014; Ng et al., 2019). Nonetheless, the relative infrequency of these responses compared to more sympathetic responses suggests that perceived prevalence of these suspicions may be inflated. However, replication is needed to see if these attributions are more prevalent among clinical providers.

There also arose a distinct dichotomy in regard to participants’ views on opioids and drug seeking within themselves relative to others. Participants discussed purposely under-reporting their pain to avoid being prescribed opioids, yet conversely often suggested that others might over-report their pain for the sole reason of obtaining drugs (for purposes other than pain relief). This highlights a potential effect of the current stigma associated with opioids, such that patients may, in order to avoid being labeled a drug seeker, receive inadequate treatment due to their under-reporting (Vallerand and Nowak, 2010; Brooks et al., 2015). For others, under-reporting pain to avoid prescription opioids reflected a preference for non-pharmacological treatments of pain, which may be overlooked by healthcare providers.

Limitations of the present study include the use of a sample whose recent and overall experience with pain is unknown, and reliance on retrospective evaluation of prior pain reporting motivations. The method of data collection was also limited by open-ended questions presented in an online setting. Structured in-person interviews may have been able to clarify some of the less detailed responses with further questioning. However, the format used allowed for a greater population to be sampled, both in terms of numbers and in representativeness. Online sampling increased the diversity our sample and thus anticipated generalizability of responses. Our combined samples including adults of all ages, from across the United States, and 35.5% of participants were Latinx-American – a demographic under-represented (and often entirely unrepresented) in the pain literature. Furthermore, whereas previous studies often target patients diagnosed with a specific disorder, participants here describe a diverse array of pain experiences contributing to pain rating modulation. The anonymity of the study may have also allowed participants to be more forthcoming about their pain reporting experiences that they may have been unwilling to or uncomfortable about sharing with an interviewer. The limited number of participants providing reasons for why others might neither over- or under-report their pain may have been due to the difference in the wording of the self- vs. other-directed questions. The self- question directed participants to reflect on their own previous experiences with pain reporting, particularly asking them *if* they ever had over- or under-reported; as such, participants were able to disclose that they had done neither of those. The other-oriented question, however, asked participants to provide thoughts on why others may over- or under-report their pain, which may have unintentionally led them to only consider these two options. Finally, the order of presentation of the self and other questions was fixed, which may have inflated the similarity of responses due to anchoring. Participants may have projected their own experiences onto those of imagined others, rather than considering other factors that might contribute to different pain experiences (Todd et al., 2016).

The results of this study have several clinical implications. Ultimately, patient modulation of pain ratings and provider discount of patient pain ratings may result in inadequate treatment. Under-reporting pain has been indicated as a barrier to effective pain assessment and treatment (Kaye et al., 2014), which in turn results in increased suffering and less satisfaction with medical care (Sherwood et al., 2000; Berglund et al., 2012). Over-reporting may lead to unnecessary procedures or over-treatment, which have shown to contribute to an increased burden both on patient health outcomes as well as the health care system (Deyo et al., 2009). Provider skepticism of patient pain reports may paradoxically lead to greater patient modulation of pain reports. Present findings indicate that improving patient-provider trust – which has known benefits for both members of these dyads (Matthias et al., 2010; Haverfield et al., 2018) – may be fundamental to decreasing modulation of pain reports (and the stress of managing multiple concerns underlying modulation), potentially increasing the utility of numerical pain ratings, and more generally optimizing pain outcomes. The attribution errors revealed in the current sample related to perceptions of the motivations of others likely emerge in clinical assessment contexts as well, and should be recognized as a potential contributing factor to provider skepticism. Finally, the far-reaching effects of pain stigma – even outside the context of chronic pain – are compelling and clinically relevant. For example, widely shared cultural conceptions of pain as weakness influence pain reporting and interpretation. Several narratives admonish that the provision and interpretation of pain reports are situated within sociocultural and structural contexts – thus potentially influenced by stigma, stereotypes, and bias.

## Data Availability Statement

The raw data supporting the conclusions of this article will be made available by the authors, without undue reservation.

## Ethics Statement

The studies involving human participants were reviewed and approved by Texas A&M University Institutional Review Board. The participants provided electronic informed consent to participate in this study.

## Author Contributions

VM and BN contributed to study conception and design. BN collected data. BB, KW, and NN conducted data analysis under the supervision of VM. BB, KW, and NN wrote the results section. KW created the final table. BB wrote the first draft of the manuscript. All authors edited the manuscript and table, have read the final draft, and have approved submission.

## Conflict of Interest

The authors declare that the research was conducted in the absence of any commercial or financial relationships that could be construed as a potential conflict of interest.

## Footnotes

1 https://www.cloudresearch.com/
